# Effects of NRG1 Polymorphisms on Hirschsprung’s Disease Susceptibility: A Meta-analysis

**DOI:** 10.1038/s41598-017-10477-w

**Published:** 2017-08-30

**Authors:** Meng Jiang, Changli Li, Guoqing Cao, Dehua Yang, Xi Zhang, Li Yang, Shuai Li, Shao-tao Tang

**Affiliations:** 1 Department of Pediatric Surgery, Union Hospital, Tongji Medical College, Huazhong University of Science and Technology, Wuhan 430022, China; 2 Department of Geratology, Hubei Provincial Hospital of Integrated Chinese and Western medicine, 11 Lingjiaohu Avenue, Wuhan 430015, Hubei Province China

## Abstract

Substantial resources have been devoted to evaluate the relationship between NRG1 variants rs7835688 and rs16879552 and Hirschsprung’s Disease (HSCR) but no consistency exists. This meta-analysis aimed to assess the association between the two SNPs and HSCR. PubMed, EMBASE, and Chinese Biological Medicine databases were searched for studies potentially eligible up to March, 2017. The summary odds ratios (ORs) with 95% CIs were calculated from different genetic models. Nine case-control studies (8 for both and 1 for rs16879552 only) involving 1984 HSCR patients and 4220 controls were identified. The combined results showed a significant association between HSCR risk and rs7835688 in all genetic models (per-allele model: OR = 1.66, 95% CI = 1.35–2.05; *P* = 1.940E-06). Rs16879552 was significantly associated with HSCR in per-allele (OR = 1.50, 95% CI = 1.27–1.76; *P* = 1.087E-06), additive and recessive model, except for dominant model. Stratified analysis by ethnicity showed that rs7835688 and rs16879552 were only causative for Asians, but not risk locus for Caucasians. Furthermore, pooled data based on segment length indicated that individuals with rs7835688 experienced a significantly higher risk for short-segment HSCR in all genotypes; but rs16879552 was only found to be associated with long-segment HSCR/ total colonic aganglionosis at the allele level.

## Introduction

As a congenital malformation of the lower gastrointestinal tract, Hirschsprung’s disease (HSCR) can be attributed to the migration of the neural crest cells (NCCs) been disrupted during embryonic development. This disorder leads to an absence of enteric ganglia in the submucosal and myenteric plexuses along a variable length of the gut which produces a functional intestinal obstruction^1^. According to the extent of the affected bowel, HSCR can be classified as short segment (S-HSCR: 80%, the aganglionic segment does not extend beyond the upper sigmoid), long-segment (L-HSCR: 15%, the aganglionosis extends to the splenic flexure or transverse colon) or total colonic aganglionosis (TCA: 5%, the aganglionosis extending from the anus to at least the ileocecal valve). The incidence of the disease has a significant racial and gender variation, and the highest morbidity is found among Asians (2.8 per 10,000 live births)^2^. Moreover, HSCR can be either sporadic or familial.

Several genes, such as RET^3, 4^, EDNRB^5^, END3^6^, GDNF^7^, PHOX2B^8, 9^ and SOX10^10^ have been found to be responsible for HSCR, implying that this disease has a complicated genetic etiology. In 2009, a genome-wide association study (GWAS) identified a new HSCR causative gene–NRG1, which was first confirmed as a susceptibility locus for HSCR in Chinese^11^. Within the NRG1 region, the single nucleotide polymorphisms (SNPs) rs7835688 (G > C) and rs16879552 (T > C) showed the strongest overall associations with HSCR, yielding odds ratios (OR) of 1.98 [CI_95%_: (1.59, 2.47), *p* = 1.12 × 10^−9^] and 1.68 [CI_95%_: (1.40, 2.00), *p* = 1.8 × 10^−6^], respectively, under an additive model. As is known, NRG1 and the ErbB family of tyrosine kinase receptors are vital molecular regulators for the NCCs’ development^12^. Loss-of-function of ErbB2 signaling in the colonic epithelial cells could lead to postnatal colonic aganglionosis in mice, for the maintenance of the enteric nervous system (ENS) is dependent on the survival factors induced by NRG1–ErbB2 interaction^13^.

Up to now, several case control studies have been conducted to investigate the association between rs7835688 and rs16879552 variants and HSCR risk^14–16^, but the results are still controversial due to the inconsistency among these studies. Recently, a research consisting of 115 HSCR patients and 117 unaffected controls in Han Chinese reported that there was no evidence of genetic association between HSCR and the two SNPs, at either allele or genotype level^17^. This is partially inconsistent to another study conducted by our team^18^. Our results demonstrated that individuals with the risk allele of rs7835688 C had an increased risk of HSCR at both allele and genotype level, whereas no genetic interaction was found between HSCR and rs16879552 under all genotypes.

To our knowledge, no quantitative reviews or meta-analysis of the literature on the association between rs7835688 and rs16879552 and HSCR have been conducted up to now. Besides, meta-analysis could reduce the risk of random error and obtain a precise prediction for the major effect through combining data from all eligible researches. In view of the accumulated data, we performed this meta-analysis to provide the evidence for the implication of NRG1 rs7835688 and rs16879552 polymorphisms in the HSCR susceptibility.

## Results

### Search Findings and Study Characteristics

**Figure 1:**
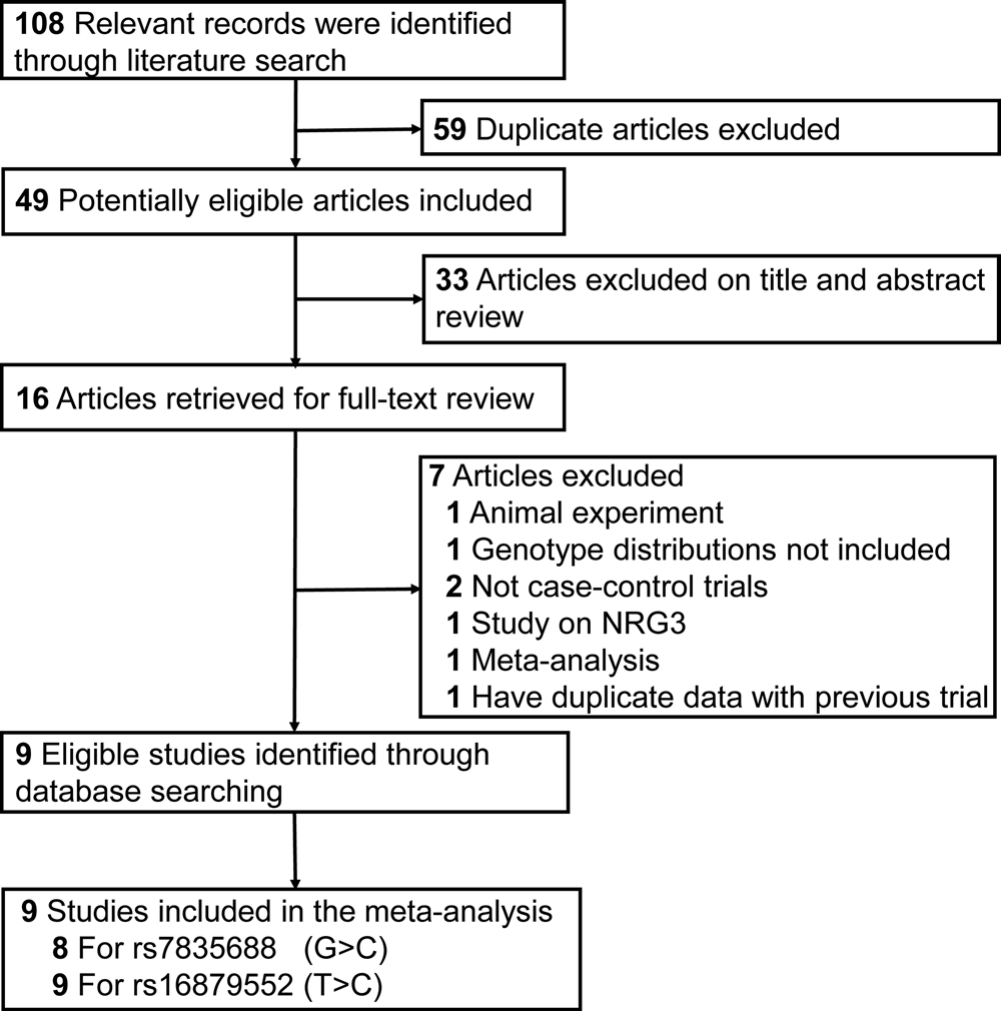
Selection of studies included in the Meta-analysis.

The process of study selection is shown in Fig. 1, which was conducted according to the PRISMA guideline for systematic review^19^. A total of 108 papers were identified after an initial search strategy from the databases. After the removal of 59 duplicate articles, 49 articles were considered of potential relevance. In total, 16 articles were retrieved for full-text review, 9 of which met our inclusion criteria^11, 14, 16–18, 20–23^. Of these 8 articles for rs7835688^11, 14, 16–18, 21–23^ and 9 articles for rs16879552^11, 14, 16–18, 20–23^ were included in the final analysis. The Table 1 shows the main characteristics of included studies. For rs7835688, the distribution of genotypes in the controls was consistent with HWE in 5 studies^16–18, 21, 22^. As for rs16879552, 4 studies^16, 17, 21, 22^ were satisfied with the HWE except one^18^. For the rest 4 studies^11, 14, 20, 23^, the authors pointed out that the genotype distribution in the controls for rs7835688 and rs16879552 didn’t violate HWE, but the exact data was not given. Of all of the studies included, 7 studies involved Asians^11, 14, 17, 18, 20–22^, 2 studies investigated Caucasians^16, 23^. All studies followed a case–control design, 2 of them were GWAS^11, 20^; 5 studies used population-based controls^14, 16, 20, 22, 23^, and 4 studies used hospital-based controls^11, 17, 18, 21^. The quality score of the included studies ranged from 9 to 11 (Table 1 and Table S1).

**Table 1:** The basic information and distribution of alleles and genotypes of rs7835688 and rs16879552.

First Author	Country	Ethnicity	Sample size		Genotype in cases			Genotype in controls			Cases		Controls		HWE P value	Source of Control	Genotyping method	Quality Score
rs7835688 G > C			Cases	Controls	CC	CG	GG	CC	CG	GG	C	G	C	G				
Garcia-Barcelo 2009	China	Asian	370	853	25	136	209	NA	NA	NA	186	554	249	1457	YES	H-B	Sequenom and SNP GeneChip; Affymetrix	10
Tang 2011	China	Asian	343	359	NA	NA	NA	NA	NA	NA	178	508	108	610	YES	P-B	TaqMan	10
Phusantisampan 2012	Thailand	Asian	68	119	13	26	29	8	43	68	52	84	59	179	0.74	P-B	TaqMan and PCR-RFLP	11
Luzon-Toro 2012	Spain	Caucasian	207	150	NA	NA	NA	NA	NA	NA	131	137	114	154	YES	P-B	TaqMan	10
Gunadi 2014	Indonesia	Asian	60	114	10	23	27	5	39	70	43	77	49	179	0.88	H-B	TaqMan	9
Kapoor 2015	USA	Caucasian	353	627	84	178	91	135	322	170	346	360	592	662	0.45	P-B	TaqMan	9
Li 2017	China	Asian	97	113	7	38	52	5	32	76	52	142	42	184	0.50	H-B	TaqMan	11
Yang 2017	China	Asian	362	1448	49	120	193	70	489	889	218	506	529	2367	0.79	H-B	TaqMan	10
**rs16879552 T > C**					**CC**	**CT**	**TT**	**CC**	**CT**	**TT**	**C**	**T**	**C**	**T**				
Garcia-Barcelo 2009	China	Asian	371	850	97	186	87	NA	NA	NA	380	360	667	1039	YES	H-B	Sequenom and SNP GeneChip; Affymetrix	10
Tang 2011	China	Asian	343	359	NA	NA	NA	NA	NA	NA	350	336	273	445	YES	P-B	TaqMan	10
Phusantisampan 2012	Thailand	Asian	68	119	43	20	5	54	45	20	106	30	153	85	0.054	P-B	TaqMan and PCR-RFLP	11
Luzon-Toro 2012	Spain	Caucasian	207	150	NA	NA	NA	NA	NA	NA	267	1	262	6	YES	P-B	TaqMan	10
Kim 2014	Korea	Asian	123	432	N A	NA	NA	NA	NA	NA	111	135	295	569	YES	P-B	Sequenom and SNP GeneChip; Illumina	11
Gunadi 2014	Indonesia	Asian	60	118	40	18	2	61	52	5	98	22	174	62	0.14	H-B	TaqMan	9
Kapoor 2015	USA	Caucasian	354	631	334	19	1	586	44	1	687	21	1216	46	0.85	P-B	TaqMan	9
Li 2017	China	Asian	96	113	20	49	27	16	56	41	89	103	88	138	0.65	H-B	TaqMan	11
Yang 2017	China	Asian	362	1448	96	128	138	323	560	565	320	404	1206	1690	7.61E-15	H-B	TaqMan	10

Abbreviations: HWE, Hardy-Weinberg equilibrium; NA, not applicable; YES, studies have already pointed out that the data was HWE, but the data was not applicable; P-B, population-based study; H-B, hospital-based study; PCR-RFLP, PCR-restriction fragment length polymorphism.

### Association between rs7835688 and risk for HSCR

**Figure 2:**
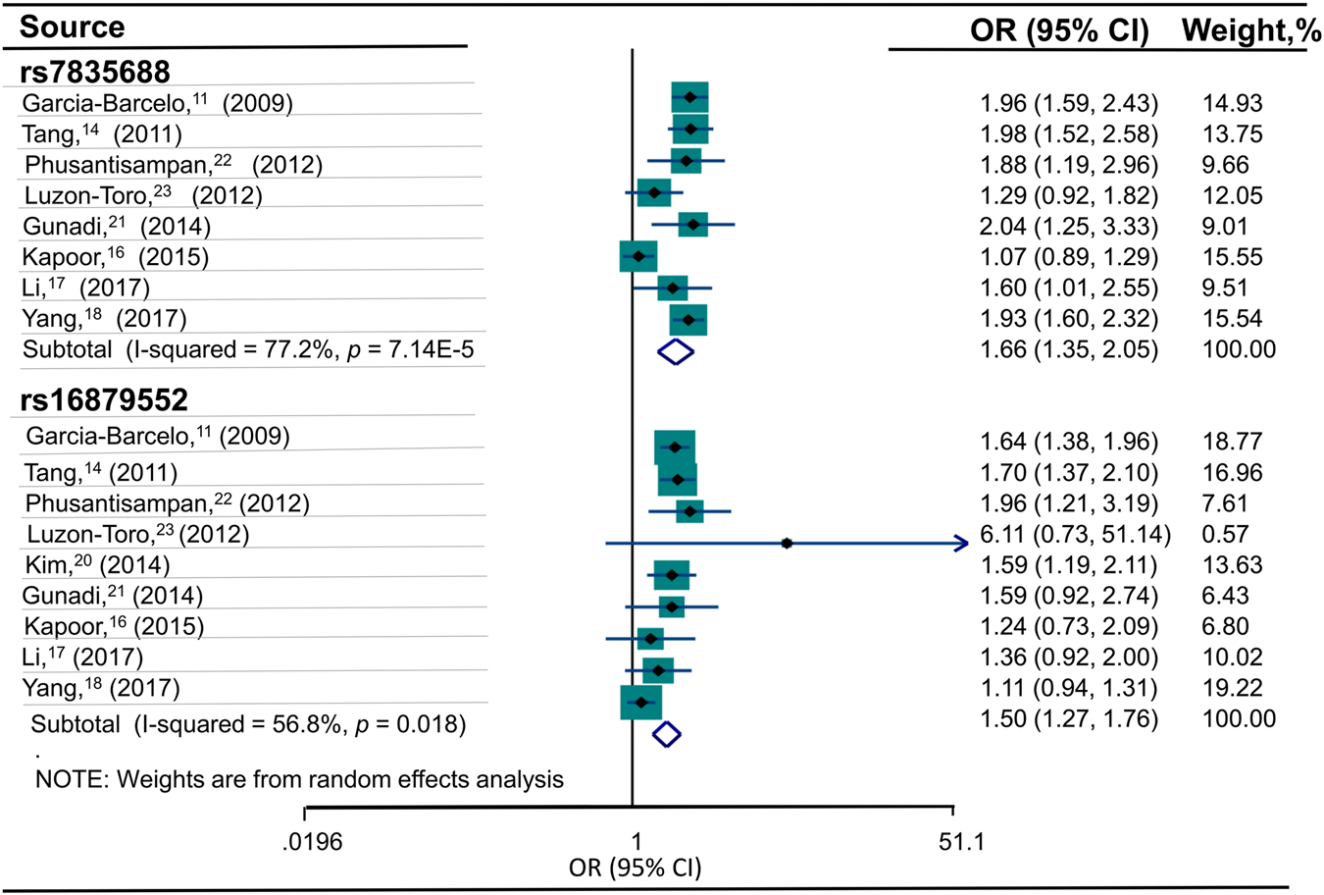
Forest plot of allele comparison for association between NRG1 variants and HSCR. HSCR indicates Hirschsprung’s Disease. The sizes of the squares are proportional to study weights. Diamond markers indicate pooled effect sizes.

There were 8 studies^11, 14, 16–18, 21–23^ including a total of 1,860 cases and 3,783 controls reported an association between rs7835688 and HSCR risk. Overall, the frequency of the C allele was 33.7% in HSCR and 23.1% in the controls. The Caucasian population bears a higher frequency of the C allele (49.0% cases vs 46.4% controls), followed by the Asian (28.0% cases vs 20.8% controls) population. The distribution of the rs7835688 genotypes and alleles is presented in Table 1. Strong evidence of an association between the rs7835688 and HSCR risk was found under the homozygous model of CC vs GG (OR = 2.63, 95% CI = 1.34–5.18, *P* = 5.170E-03; *I* ^2^ = 80.5%, *P* = 3.977E-04) (Table 2). We also found a significant association under per-allele model (OR = 1.66, 95% CI = 1.35–2.05, *P* = 1.940E-06; *I* ^2^ = 77.2%, *P* = 7.141E-05) (Fig. 2 and Table 2), dominant model (OR = 1.60, 95% CI = 1.20–2.13, *P* = 1.359E-03; *I* ^2^ = 58.2%, *P* = 4.838E-02), and recessive model (OR = 2.57, 95% CI = 1.17–5.66, *P* = 1.872E-02; *I* ^2^ = 87.7%, *P* = 1.430E-06) (Table 2). According to the stratified analysis by ethnicity, a robust association was found between rs7835688 and HSCR risk among the Asian population under all genetic models, with no evidence of heterogeneity. In contrast, the association between rs7835688 and HSCR was not significant in Caucasian population (Table 3). Furthermore, subgroup analysis by HSCR segment length indicated that patients with rs7835688 polymorphism were more easily develop into S-HSCR than L-HSCR/TCA (Table 4).

**Table 2:** Association between NRG1 polymorphisms and HSCR risk.

Gene polymorphism	Number of studies	Comparison model	Test of association			Test of heterogeneity			P value for publication bias	
			OR	95%CI	P value	Q	P value	I^2^ (%)	Begg’s test	Egger’s test
rs7835688 (G > C)	5	CC vs GG	2.63	1.34–5.18	5.170E-03	20.5	3.977E-04	80.5	—	—
	5	CC vs CG + GG	2.57	1.17–5.66	1.872E-02	32.62	1.430E-06	87.7	—	—
	5	CC + CG vs GG	1.60	1.20–2.13	1.359E-03	9.57	4.838E-02	58.2	—	—
	8	C vs G	1.66	1.35–2.05	1.940E-06	30.67	7.141E-05	77.2	0.711	0.652
rs16879552 (T > C)	5	CC vs TT	1.38	1.03–1.83	2.912E-02	4.10	3.926E-01	2.4	—	—
	5	CC vs CT + TT	1.74	1.18–2.57	5.548E-03	11.29	2.354E-02	64.6	—	–
	5	CC + CT vs TT	1.21	0.90–1.63	1.979E-01	4.53	3.395E-01	11.6	—	—
	9	C vs T	1.50	1.27–1.76	1.087E-06	18.51	1.770E-02	56.8	0.917	0.325

**Table 3:** Subgroup analyses by ethnicity.

Gene polymorphism	Comparison	Asian				Caucasian			
	Model	OR (95%CI) (N)	P value^a^	I^2^ (%)	P value^b^	OR (95%CI) (N)	P value^a^	I^2^ (%)	P value^b^
rs7835688	CC vs GG	3.57 (2.55–4.99) (4)	1.289E-13	0	7.435E-01	1.16 (0.80–1.69) (1)	4.289E-01	—	—
	CC vs CG + GG	4.01 (2.88–5.58) (4)	1.898E-16	0	4.435E-01	1.14 (0.83–1.55) (1)	4.140E-01	—	—
	CC + CG vs GG	1.87 (1.54–2.27) (4)	2.490E-10	0	9.951E-01	1.06 (0.80–1.44) (1)	6.501E-01	—	—
	C vs G	1.93 (1.72–2.16) (6)	2.519E-30	0	9.807E-01	1.12 (0.95–1.32) (2)	1.692E-01	0	3.522E-01
rs16879552	CC vs TT	1.51 (1.02–2.23) (4)	3.997E-02	19.4	2.929E-01	0.57 (0.04–9.14) (1)	6.913E-01	—	—
	CC vs CT + TT	1.91 (1.15–3.16) (4)	1.183E-02	72.4	1.247E-02	1.28 (0.74–2.21) (1)	3.698E-01	—	—
	CC + CT vs TT	1.30 (0.90–1.88) (4)	1.661E-01	29.8	2.333E-01	0.56 (0.03–8.99) (1)	6.824E-01	—	—
	C vs T	1.50 (1.27–1.78) (7)	2.568E-06	63.4	1.177E-02	1.96 (0.47–8.13) (2)	3.559E-01	51.8	1.498E-01

N, Number of studies; ^a^ *P* value refers to the overall effect; ^b^ *P* value refers to the heterogeneity.

**Table 4:** Subgroup analyses by HSCR segment length.

Gene polymorphism	Comparison	S-HSCR				L-HSCR/TCA			
	model	OR (95%CI) (N)	P value^a^	I^2^ (%)	P value^b^	OR (95%CI) (N)	P value^a^	I^2^ (%)	P value^b^
rs7835688	CC vs GG	3.53 (2.41–5.15) (2)	7.563E-11	0	7.704E-01	1.17 (0.21–6.40) (1)	8.569E-01	—	—
	CC vs CG + GG	1.84 (1.25–2.71) (2)	2.132E-03	0	5.741E-01	0.93 (0.16–5.28) (1)	9.284E-01	—	—
	CC + CG vs GG	1.93 (1.55–2.40) (2)	5.549E-09	0	5.865E-01	1.26 (0.61–2.64) (1)	5.328E-01	—	—
	C vs G	1.95 (1.69–2.24) (3)	7.387E-21	0	9.852E-01	1.60 (1.00–2.57) (2)	5.162E-02	36.2	2.105E-01
rs16879552	CC vs TT	1.25 (0.94–1.66) (2)	1.197E-01	0	5.433E-01	2.31 (0.79–6.72) (1)	1.258E-01	—	—
	CC vs CT + TT	1.28 (1.00–1.65) (2)	5.455E-02	0	6.428E-01	1.65 (0.67–4.10) (1)	2.773E-01	—	—
	CC + CT vs TT	1.06 (0.85–1.32) (2)	6.211E-01	0	6.352E-01	1.82 (0.81–4.08) (1)	1.450E-01	—	—
	C vs T	1.32 (0.99–1.77) (3)	6.197E-02	78.1	1.035E-02	1.64 (1.21–2.24) (2)	1.579E-03	0	6.400E-01

Abbreviations: HSCR, Hirschsprung’s Disease; N, Number of studies; S-HSCR, short-segment Hirschsprung’s Disease; L-SHCR/TCA: long-segment Hirschsprung’s Disease /total colonic aganglionosis. ^a^ *P* value refers to the overall effect; ^b^ *P* value refers to the heterogeneity.

### Association between rs16879552 and risk for HSCR

Overall, 9 studies with 1,984 cases and 4,220 controls analyzed the rs16879552 and risk of HSCR^11, 14, 16–18, 20–23^. The frequency of the risk C allele was 61.0% in the cases and 50.1% in the controls in total. In Asian subjects, the frequency of the C allele was 48.5% in cases and 40.5% in controls, and these values are lower than those found in the Caucasian population (97.7% cases vs 96.6% controls). After analyzing the relationship between the C allele and the risk of HSCR, we found no significant association between rs16879552 polymorphism and HSCR under dominant model of CC + CT vs TT (OR = 1.21, 95% CI = 0.90–1.63, *P* = 1.979E-01; *I* ^2^ = 11.6%, *P* = 3.395E-01) (Table 2). However, significant association was observed under per-allele model (OR = 1.50, 95% CI = 1.27–1.76, *P* = 1.087E-06; *I* ^2^ = 56.8%, *P* = 1.770E-02) (Fig. 2 and Table 2), additive model and recessive model (Table 2). A further subgroup analysis by ethnicity showed no obvious association between the rs16879552 polymorphism and HSCR in Caucasian subjects, while a significant association was observed in the Asian population under the per-allele, additive and recessive model (Table 3). As for stratified analysis based on the type of HSCR, the association between the rs16879552 and HSCR was only significant in the L-HSCR/TCA at allele level (Table 4).

### Sensitivity Analysis

**Figure 3:**
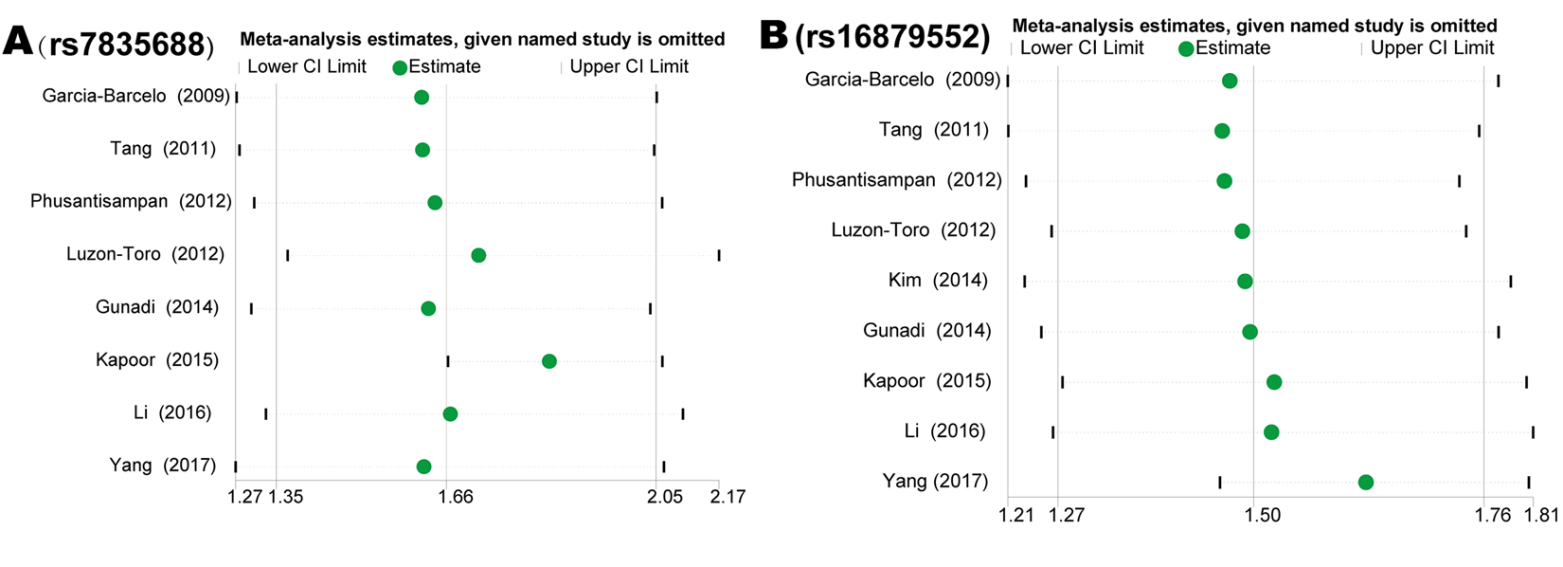
Results of sensitivity analysis under per-allele model. The green dots and lines indicate the odds ratios (ORs) and their 95% confidence intervals (CIs), given named study is omitted.

We performed sensitivity analysis under per-allele model to evaluate the influence of a specific publication on the overall estimate. The corresponding pooled ORs with 95% CIs for rs7835688 and rs16879552 were not substantially altered before and after omitting any single study at a time, implying that that our results were stable and reliable (Fig. 3). This analysis also revealed that one study, by Kapoor *et al*.^16^, was the main source of heterogeneity for rs7835688. As is shown in Fig. 3A, after omitting this paper, the lower 95% CI Limit (1.67) was larger than the overall OR (1.66), and the *I* ^2^ decreased from 77.2% (*P* = 7.141E-05) to 0.0% (*P* = 0.477). However, the pooled OR after removing this study was 1.85 (95% CI, 1.67–2.06), which was not deviated from the total estimate substantially. Furthermore, after exclusion of the article (by Yang *et al*.) deviating from HWE in the controls for rs16879552^18^, the result of the relationship was not influenced significantly (Fig. 3B). The sensitivity analysis also indicated that our results were robust under the other three genotype models for both rs7835688 and rs16879552 (data not shown).

### Publication Bias

**Figure 4:**
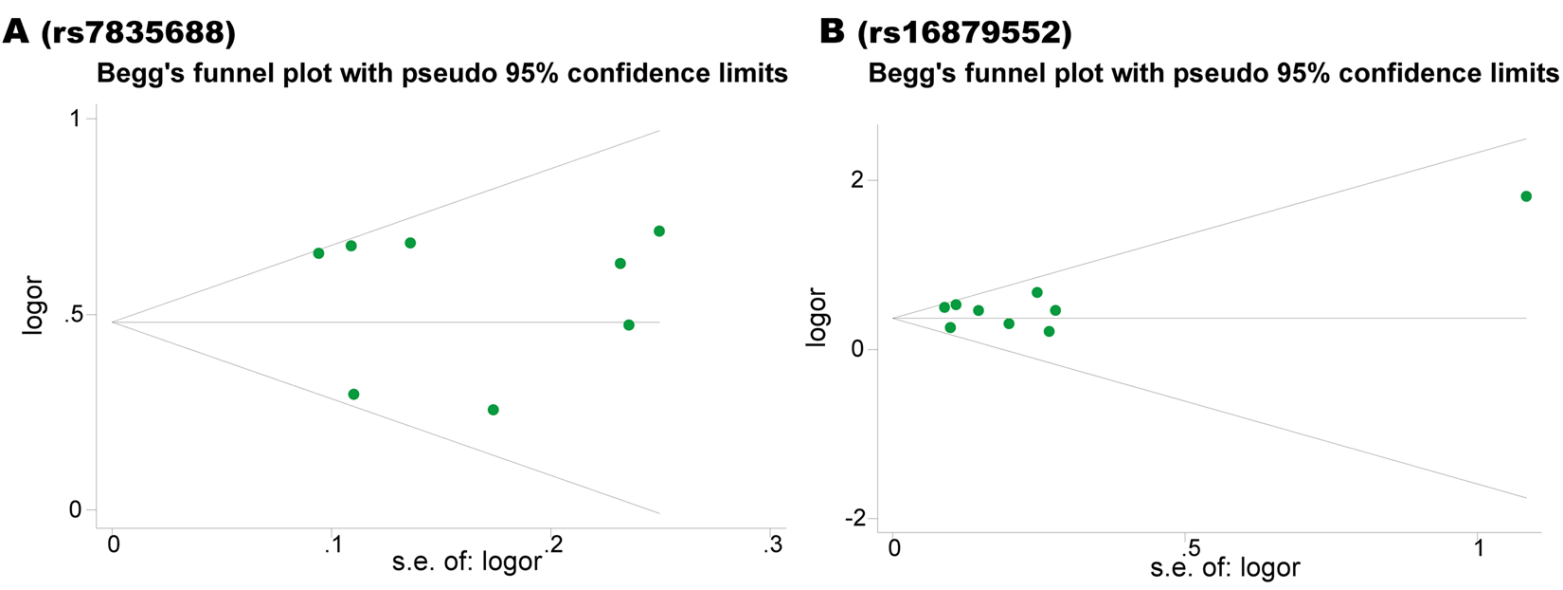
Begg’s funnel plot of publication bias for rs7835688 and rs16879552. The horizontal line in the funnel plot indicates the random-effects summary OR, while the sloping lines indicate the expected 95% confidence intervals for a given standard error, assuming no heterogeneity between studies. OR, odds ratio; s.e., standard error.

Begg’s funnel plot was conducted under per-allele model to evaluate the publication bias of the retrieved studies. As is shown in Fig. 4, the shape of funnel plots for both rs7835688 and rs16879552 were symmetrical. Additionally, neither the Begg’s tests (rs7835688: *P* = 0.711; rs16879552: *P* = 0.917) nor the Egger’s tests (rs7835688: *P* = 0.652; rs16879552: *P* = 0.325) supported the existence of publication bias (Table 2).

## Discussion

In the current meta-analysis, we have provided a systematic evaluation of the association between the NRG1 rs7835688 and rs16879552 polymorphisms and HSCR susceptibility, including its subtypes S-HSCR and L-HSCR/TCA. The combined results of included studies suggested that rs7835688 polymorphism exerted a significant influence on HSCR risk in Asians. Given that there were only 2 studies included in the subgroup analysis and the heterogeneity was not minimal, our meta-analysis did not demonstrate a definite association between rs7835688 polymorphism and HSCR in Caucasian population. Additionally, further analysis showed that individuals with rs7835688 polymorphism experienced a significantly higher risk for S-HSCR. With respect to rs16879552, we found a modest association in Asian patients rather than Caucasians. Subgroup analysis based on the HSCR segment length revealed a relationship with L-HSCR/TCA at the allele level.

NRG1 was first identified as a susceptibility locus for HSCR by Garcia-Barcelo and colleagues in Chinese^11^. Actually, this result has a biological plausibility and could be easily understood in the light of the known function of NRG1 that is implicated in the development of the ENS^13, 24^. The NRG1/ErbB system promotes neuronal survival and plays an important role in the maintenance of the ENS^25, 26^. The association between rs7835688 and rs16879552 variants and HSCR had been repeatedly verified in Asians^14, 20, 22^, however, the conclusions remained controversial because of the inconsistent findings from different ethnicity^16, 23^ or even from different country or region within Asia^17, 18, 21^. In Thai patients, Phusantisampan *et al*.^22^ found a comparable association with the Chinese study^11^ between both rs7835688 and rs16879552 polymorphisms and HSCR susceptibility. However, in a later study in Korean patients, only a nominal relevance at rs16879552 was shown^20^. Recently, both Li’s^17^ and our study^18^ uncovered that the risk allele rs7835688 C predisposed the hosts more susceptible to S-HSCR, but did not find an association between rs16879552 C and the risk of HSCR. Remarkably, another two studies from American and Spain revealed that neither rs7835688 nor rs16879552 was involved in Caucasian HSCR^16, 23^.

After been identified as a HSCR causative locus, several functional studies have been initiated to explore the genotype-phenotype association between NRG1 and HSCR. Garcia-Barcelo and colleagues found that the expression of NRG1 was decreased in the aganglionic bowel^11^. A later study reported that the overall NRG1 expression in the intestine did not differ between HSCR patients and controls^14^. However, this research only took full-thickness tissues from ganglionic bowel of the patients and compared it with the controls. Even though no association between rs7835688 and rs16879552 and HSCR risk was detected, another three novel variants (M111T, M139I and R438H) of NRG1 were found to be causal mutations for HSCR in Caucasian population^23^. Immunocytochemistry illustrated a different distribution of the NRG1 proteins in the cytoplasmic organelles between wildtype and mutants (M111T, M139I and R438H) in COS7 cell line. Besides, all three mutants showed a substantial lower protein expression. The results suggested that NRG1 would be associated with HSCR not only in Asian but also in Caucasian population. In contrast to the previous reports, aberrant high expression of NRG1 in aganglionic bowel of HSCR patients was observed in another study, but how this discrepancy happened was not clear^27^. Mounting evidence have vindicated the role of NRG1 in the HSCR pathology, however, the underlying mechanisms were still largely unknown. Further research into the pathogenesis of HSCR is needed.

The ethnicity, type of HSCR and sex distribution might serve as confounders to influence the effect size, so we stratified data from the included studies to evaluate the association between NRG1 variants and HSCR risk in confounder-matched groups. In ethnicity-based studies, our results showed that rs7835688 and rs16879552 related to HSCR appeared to be Asian-specific. Moreover, when the data were stratified by segment length, a robust association was found between rs7835688 and risk of S-HSCR, in all genotypes with no heterogeneity (*I* ^2^ = 0, *P* > 0.10). But quite on the contrary, we just identified a marginal association between rs16879552 and L-HSCR/TCA only in allelic association analysis. It is necessary to point out that the HSCR type-based subgroup analysis was limited to Asians due to no available data was provided in the other two studies about Caucasians^16, 23^. The sensitivity analysis demonstrated that the literature by Kapoor *et al*.^16^ was the main source of heterogeneity for rs7835688. The heterogeneity was significantly decreased (*I* ^2^ = 0.0%) after excluding this study. Nevertheless, the summary OR did not changed essentially, supporting the stability of the pooled results.

The polymorphic variance of NRG1 could also be attributed to the gender difference. Unfortunately, only one of the studies in our research provided detailed information of genotype distributions in males and females^11^. In this research, no significant allele frequency difference was observed by gender, for both rs7835688 C (24.48% versus 27.44%, *P* = 0.44) and rs16879552 C (51.22% versus 51.83%, *P* = 0.89).

As a meta-analysis, some intrinsic limitations need to be acknowledged. First, significant heterogeneity was observed across studies for the association between the two SNPs and HSCR risk, which might result from differences in study quality, study populations, and ratios of the subgroups (specifically short-segment patients). However, despite moderate to high heterogeneity existed for the overall effect, in the ethnicity- and segment length- based subgroup analysis low heterogeneity was detected in most of the genotypes. Second, most of the study subjects came from Asian ancestry, and the Caucasian subgroup was very limited in our meta-analysis. Thus, potential publication bias and selective bias may have occurred. Third, residual confounding is still possible since HSCR is a multifactor malformation, gene-environment and gene-gene interactions should be considered. Finally, the sample size of L-HSCR/TCA in this meta-analysis was not big enough to reach a strong statistical power for making a definite conclusion about the risk of rs7835688 and rs16879552 for these patients.

Despite the limitations, we believe that our meta-analysis have provided accumulated and useful evidence for the role of NRG1 in HSCR. First, the sample size of each single study in our meta-analysis was not large enough to achieve a definite association between the NRG1 polymorphisms (rs7835688 and rs16879552) and HSCR risk, but the pooled OR calculated from the 8 or 9 studies significantly increased the statistical power. This is essential in genetic association studies to obtain adequate statistic power^28^. Second, no significant publication bias was detected in this meta-analysis, and the results were proved to be stable by the sensitivity analysis. Furthermore, this meta-analysis was in line with our previous fine mapping of the two SNPs by showing that rs7835688 played a role in predisposition to S-HSCR^18^.

## Conclusions

Our analysis provides substantial evidence that NRG1 rs7835688 and rs16879552 are significantly associated with increased risk of HSCR. This finding expands the number of confirmed HSCR susceptibility loci. The NRG1 locus may represent another pathway in the pathogenesis of HSCR and could lead to insights regarding ways to modify the risk of HSCR.

## Materials and Methods

### Search strategy

We searched the PubMed, EMBASE, and Chinese Biological Medicine data bases until March, 2017, using the search terms [“NRG1” OR “NRG 1” OR “neuregulin 1” OR “neuregulin-1” OR “neuregulin1”] and [“Hirschsprung’s Disease” OR “Hirschsprung Disease” OR “HSCR” OR “HD”] to identify eligible studies that investigating the association between NRG1 SNPs and HSCR risk. In addition, the reference lists of the selected articles were hand checked to find other relevant publications that might be missed in the initial search strategy. We imposed no language or year restrictions on the search strategy.

### Study Selection

Two of us independently assessed the retrieved studies (M.J. and C.-L.L.). Potentially relevant studies were selected based on the following inclusion criteria: (1) studies could be defined as case–control or cohort study; (2) studies in which the diagnosis of HSCR was clear (the diagnosis was based on pathological sections); (3) studies had examined the associations between the NRG1 SNPs (rs7835688 or rs16879552) and HSCR; (4) the genotype data in case and control groups could be collected; (5) the cases and controls were recruited from a population with the same ethnic background. Studies with duplicated data or no available data were excluded. In the case of different articles related to the same patient population, only the reports with the highest number of cases were included.

### Data extraction

Two reviewers (G.-Q.C. and D.-H.Y.) extracted the data from all eligible articles independently, according to the inclusion and exclusion criteria. Any disagreements were resolved by discussion between the two reviewers. The following data were extracted: name of first author, year of publication, country, ethnicity of the subjects, source of control, the genotyping method, sample size, frequency of NRG1 genotypes in the cases and controls; and the *P* values for Hardy-Weinberg equilibrium (HWE) in controls. We contacted the authors of included studies if additional raw data were needed.

### Quality score assessment

Two reviewers (M.J. and L.Y.) assessed the quality of the studies independently with a checklist modified from Thakkinstian *et al*.^29^, which was based on both genetic issues and traditional epidemiologic considerations. The checklist contained 7 aspects: representativeness of cases, representativeness of controls, ascertainment of HSCR, ascertainment of controls, genotyping examination, Hardy-Weinberg equilibrium and association assessment. Total scores ranged from zero (worst) to 13 (best). Details of each item were outlined in Table S2.

### Statistical analysis

ORs with 95% confidence intervals (CI) were calculated for determining the strength of the relationship between rs7835688 and rs16879552 and HSCR. The pooled ORs for rs7835688 and rs16879552 were estimated under four genetic models, namely, per-allele model (C vs G or C vs T), an additive/homozygous model (CC vs GG or CC vs TT), a dominant model (CC + CG vs GG or CC + CT vs TT) and a recessive model (CC vs CG + GG or CC vs CT + TT), respectively. If the *P* value of HWE was not given, it was assessed by Chi-square test to analyze the genotype distribution in the control groups. In addition, we used the Cochrane Q statistic and the inconsistency index (*I* ^2^) to evaluate the heterogeneity among the retrieved studies; *P* value < 0.10 or *I* ^2^ > 50% was considered statistically significant for the heterogeneity^30^. If heterogeneity existed, we selected the random-effect model (the Dersimonian and Laird method) to calculate the pooled OR. Otherwise, the fixed effects model (the Mantel-Haenszel method) should be used if no obvious heterogeneity was detected^31^. Sensitivity analysis was performed by excluding individual studies to assess the stability of the overall OR. The publication bias was assessed using both Egger’s test and Begg’s test^32^. The visual inspection of funnel plots was also used to show the extent of publication bias^33^. Additionally, subgroup analyses were conducted by ethnicity and segment length (S-HSCR or L-HSCR/TCA). The statistical analysis was performed with STATA software version 12.0 (Stata Corp, College Station, TX). Except for heterogeneity, *P* value of < 0.05 (two tailed) was considered to be significant statistically in this report.

## Electronic supplementary material

Supplementary Information 
