# Supplementary material for: Effects of NRG1 Polymorphisms on Hirschsprung’s Disease Susceptibility: A Meta-analysis
Source: Sci Rep. 2017 Aug 30;7:9913. doi: 10.1038/s41598-017-10477-w (PMC5577043; doi:10.1038/s41598-017-10477-w)
Supplement: Supplementary file 1 — Supplementary Information [file 41598_2017_10477_MOESM1_ESM.doc]

**Effects of NRG1 Polymorphisms on Hirschsprung’s Disease Susceptibility：A Meta-analysis**

**Meng Jiang^1,+^, Changli Li^2, +^，Guoqing Cao^1,+^, Dehua Yang^1^, Xi Zhang^1^, Li Yang^1^, Shuai Li^1^, Shao-tao Tang^1,*^**

^1^ Department of Pediatric Surgery, Union Hospital, Tongji Medical College, Huazhong University of Science and Technology, Wuhan 430022, China

^2^ Department of Geratology, Hubei Provincial Hospital of Integrated Chinese and Western medicine, 11 Lingjiaohu Avenue, Wuhan 430015, Hubei Province, China

**Table S1: Quality assessment of included studies.**

| **Study** | **Representativeness of cases** | **Representativeness of controls** | **Ascertainment of HSCR** | **Ascertainment of controls** | **Genotyping examination** | **Hardy-Weinberg equilibrium** | **Association assessment** | **Total score** |
| --- | --- | --- | --- | --- | --- | --- | --- | --- |
| Garcia-Barcelo 2009 | 2 | 1 | 2 | 1 | 0 | 2 | 2 | 10 |
| Tang 2011 | 2 | 1 | 2 | 1 | 0 | 2 | 2 | 10 |
| Phusantisampan 2012 | 2 | 1 | 2 | 2 | 0 | 2 | 2 | 11 |
| Luzon-Toro 2012 | 2 | 2 | 1 | 1 | 0 | 2 | 2 | 10 |
| Kim 2014 | 2 | 2 | 2 | 1 | 0 | 2 | 2 | 11 |
| Gunadi 2014 | 1 | 2 | 2 | 1 | 0 | 2 | 2 | 9 |
| Kapoor 2015 | 2 | 1 | 2 | 0 | 0 | 2 | 2 | 9 |
| Li 2017 | 2 | 2 | 2 | 1 | 0 | 2 | 2 | 11 |
| Yang 2017 | 2 | 1 | 2 | 1 | 0 | 2 | 2 | 10 |

Abbreviation: HSCR, Hirschsprung’s Disease.

**Table S2: Scale for quality assessment of selected s**tudy.

| **Criteria** | **Score** |
| --- | --- |
| **Representativeness of cases** |  |
| Consecutive/randomly selected from case population with clearly deﬁned sampling frame | 2 |
| Consecutive/randomly selected from case population without clearly deﬁned sampling frame or with extensive inclusion/exclusion criteria | 1 |
| No method of selection described | 0 |
| **Representativeness of controls** |  |
| Controls were consecutive/randomly drawn from the  same sampling frame (ward/community) as cases | 2 |
| Controls were consecutive/randomly drawn from  a different sampling frame as cases | 1 |
| Not described | 0 |
| **Ascertainment of HSCR** |  |
| Clearly described objective criteria for diagnosis of HSCR | 2 |
| Diagnosis of HSCR by patient self-report or by patient history | 1 |
| Not described | 0 |
| **Ascertainment of controls** |  |
| Controls were ruled out HSCR. i.e., perform rectal suction biopsies or clinical followed up | 2 |
| Controls were subjects who did not report HD; no objective testing | 1 |
| Not described | 0 |
| **Genotyping examination** |  |
| Genotyping done under ‘‘blinded’’ condition | 1 |
| Un-blinded or not mentioned | 0 |
| **Hardy-Weinberg equilibrium** |  |
| Hardy-Weinberg equilibrium in control group | 2 |
| Hardy-Weinberg disequilibrium in control group | 1 |
| No checking for Hardy-Weinberg equilibrium | 0 |
| **Association assessment** |  |
| Assess association between genotypes and HSCR with appropriate statistics and adjustment for confounders | 2 |
| Assess association between genotypes and HSCR with appropriate statistics without adjustment for confounders | 1 |
| Inappropriate statistics used | 0 |

Abbreviation: HSCR, Hirschsprung’s Disease.
